# Modeling Response Time and Responses in Multidimensional Health Measurement

**DOI:** 10.3389/fpsyg.2019.00051

**Published:** 2019-01-29

**Authors:** Chun Wang, David J. Weiss, Shiyang Su

**Affiliations:** ^1^College of Education, University of Washington, Seattle, WA, United States; ^2^Department of Psychology, University of Minnesota, St. Paul, MN, United States; ^3^Department of Psychology, University of Central Florida, Orlando, FL, United States

**Keywords:** response time, hierarchical model, health measurement, multidimensional graded response model, item response theory (IRT)

## Abstract

This study explored calibrating a large item bank for use in multidimensional health measurement with computerized adaptive testing, using both item responses and response time (RT) information. The Activity Measure for Post-Acute Care is a patient-reported outcomes measure comprised of three correlated scales (Applied Cognition, Daily Activities, and Mobility). All items from each scale are Likert type, so that a respondent chooses a response from an ordered set of four response options. The most appropriate item response theory model for analyzing and scoring these items is the multidimensional graded response model (MGRM). During the field testing of the items, an interviewer read each item to a patient and recorded, on a tablet computer, the patient's responses and the software recorded RTs. Due to the large item bank with over 300 items, data collection was conducted in four batches with a common set of anchor items to link the scale. van der Linden's (2007) hierarchical modeling framework was adopted. Several models, with or without interviewer as a covariate and with or without interaction between interviewer and items, were compared for each batch of data. It was found that the model with the interaction between interviewer and item, when the interaction effect was constrained to be proportional, fit the data best. Therefore, the final hierarchical model with a lognormal model for RT and the MGRM for response data was fitted to all batches of data via a concurrent calibration. Evaluation of parameter estimates revealed that (1) adding response time information did not affect the item parameter estimates and their standard errors significantly; (2) adding response time information helped reduce the standard error of patients' multidimensional latent trait estimates, but adding interviewer as a covariate did not result in further improvement. Implications of the findings for follow up adaptive test delivery design are discussed.

## Introduction

When assessments are delivered via computer-based devices, collecting persons' response times (RTs) at the item level is straightforward. The analysis of item-level RTs on assessments has attracted substantial interest recently. For example, in personality assessments, RTs have been used to measure attitude strength (Bassili, 1996), to detect social desirability (Holden and Kroner, 1992), and to enhance criterion validity (Siem, 1996). In achievement testing, RTs have been used to evaluate the speededness of the test (Van Der Linden et al., 1999), to detect aberrant behavior (e.g., Wang and Xu, 2015; Wang et al., 2018b,c), and to design a more efficient test (Bridgeman and Cline, 2004; Van der Linden and Guo, 2008; van der Linden, 2009; Fan et al., 2012). RTs have also been used to evaluate response data quality in web-based surveys (Galesic and Bosnjak, 2009).

In the health measurement domain, response time (sometimes called reaction time) is often used to measure cognitive functioning, particularly in research on aging (e.g., Pearson, 1924; Braver and Barch, 2002; Hultsch et al., 2002; Anstey et al., 2005; Osmon et al., 2018). Similar to the speed test in educational assessments, RTs are usually collected from timed, target stimuli tasks, in which respondents are instructed to respond as quickly as possible. In this case, only RTs, not response accuracy, is of interest. For example, in a study using the United Kingdom Health and Lifestyle Survey (Cox et al., 1987; Der and Deary, 2006), person-level reaction times were examined across different age and gender groups. Another example is using RTs from a stop-signal reaction time task to study response inhibition from patients with Parkinson's disease and other brain disorders (Gauggel et al., 2004; Verbruggen et al., 2013). Despite these widespread applications of RTs, little attention has been paid to the usefulness of item-level response times as collateral information for improving measurement precision. These previous studies have primarily used scale-level, aggregated RTs, such as its mean and standard deviation. However, item-level RTs, routinely collected during computer-based assessment delivery, provide richer information. Only a recent didactic review by Osmon et al. (2018) demonstrated the advantages of examining the entire RT distribution rather than only its mean and standard deviation to understand the efficacy of mental speed assessment in clinical neuropsychology. Therefore, it was of interest to apply advanced psychometric models for item-level RTs in the assessment of reported health behaviors and evaluate if RTs help better estimate the main constructs of interest.

## Models

### Multidimensional Graded Response Model

The most appropriate measurement model for ordered polytomous responses is the graded response model (GRM; Samejima, 1969). The item response function of the unidimensional GRM model is

(1) $$\begin{array}{r} P_{jk}\left(\theta \right)=P_{jk}^{+}\left(\theta \right)-P_{j,k+1}^{+}\left(\theta \right)=\frac{{\text{e}}^{\left[Da_{j}\left(\theta -b_{jk}\right)\right]}}{1+{\text{e}}^{\left[Da_{j}\left(\theta -b_{jk}\right)\right]}}\\ -\;\frac{{\text{e}}^{\left[Da_{j}\left(\theta -b_{j,k+1}\right)\right]}}{1+{\text{e}}^{\left[Da_{j}\left(\theta -b_{j,k+1}\right)\right]}} \end{array}$$

where *P*_*jk*_(*θ*) is the probability of a randomly selected person with a latent trait *θ* selecting category *k* of item *j* (*k*−1 … *K*). $P_{jk}^{+}\left(\theta \right)$ is the boundary response function, interpreted as the probability of responding to category *k* and above for item *j* given *θ*. *a*_*j*_ is the item discrimination parameter for item *j*. *b*_*jk*_ is the boundary location parameter for item *j* in category *k* (*k* = 0, …, *K*). D = 1.7 is the normalizing constant. Because by definition, $P_{j0}^{+}\left(\theta \right)\equiv 1\text{ and }P_{jK+1}^{+}\left(\theta \right)\equiv 0$, neither *b*_*j*0_ nor *b*_*jK*+1_ are estimable parameters. Therefore, for an item with four response categories, only three boundary parameters are estimated.

When the instruments include multiple scales measuring different constructs or different aspects of the same construct (e.g., Zickar and Robie, 1999; Fraley et al., 2000; Fletcher and Hattie, 2004; Zagorsek et al., 2006; Pilkonis et al., 2014), the multidimensional extension of the GRM, namely, the MGRM (Hsieh et al., 2010; Jiang et al., 2016), is appropriate. Let ***θ*** be a vector of length *H* representing the latent traits of interest, and let *h* = 1, 2, …, *H*. Similar to the unidimensional case, $P_{j0}^{+}\left(\text{θ}\right)\equiv 1$ and $P_{j\left(K+1\right)}^{+}\left(\text{θ}\right)\equiv 0$. When the test displays a simple structure, the boundary response function takes the form of

(2) $$\begin{array}{r} P_{jk}^{+}\left(\text{θ}\right)=\frac{1}{1+\exp\left[-\text{D}a_{jh}\left(\theta_{h}-b_{jk}\right)\right]}\\ \;=\frac{1}{1+\exp\left[-\text{D}\left(a_{jh}\theta_{h}+c_{jk}\right)\right]}, \end{array}$$

assuming item *j* measures dimension *h* only so that *a*_*jh*_ is the item discrimination parameter on the *h*th dimension of item *j*. In Equation 2, *c*_*jk*_ = −*a*_*jh*_*b*_*jk*_ and this *a-c* parameterization with *D* = 1 is consistent with *flex*MIRT's (Cai, 2013) default parameterization; the *c* parameter is interpreted as the “intercept.” Equation 2 could also be modified to accommodate complex structure; for details, see Reckase (2009).

### Bivariate Models of Responses and RTs

Given that RTs carry useful collateral information about both item and person characteristics, the bivariate model of responses and RTs (Molenaar et al., 2015) was considered. The measurement model for responses was as specified in Equation 2, and the measurement model for RTs takes the form

(3) $$\ln\text{​}t_{ij}=\lambda_{j}+\;\varphi_{j}\tau_{i}-\;\varphi_{j}\rho_{d}\theta_{id}+\omega_{ij}$$

Here, *t*_*ij*_ denotes the RT of patient *i* on item *j*, τ_*i*_ is the latent speed parameter of patient *i*, λ_*j*_ and φ_*j*_ are the time-intensity and time-discrimination parameters of item *j*, and ω_*ij*_ is the residual. If the residuals are assumed to be normally distributed, then Equation 3 suggests that the response time *t*_*ij*_ follows a log-normal distribution. Other more flexible types of residuals can also be assumed if the data warrants (e.g., Wang et al., 2013a,b).

**Figure 1:**
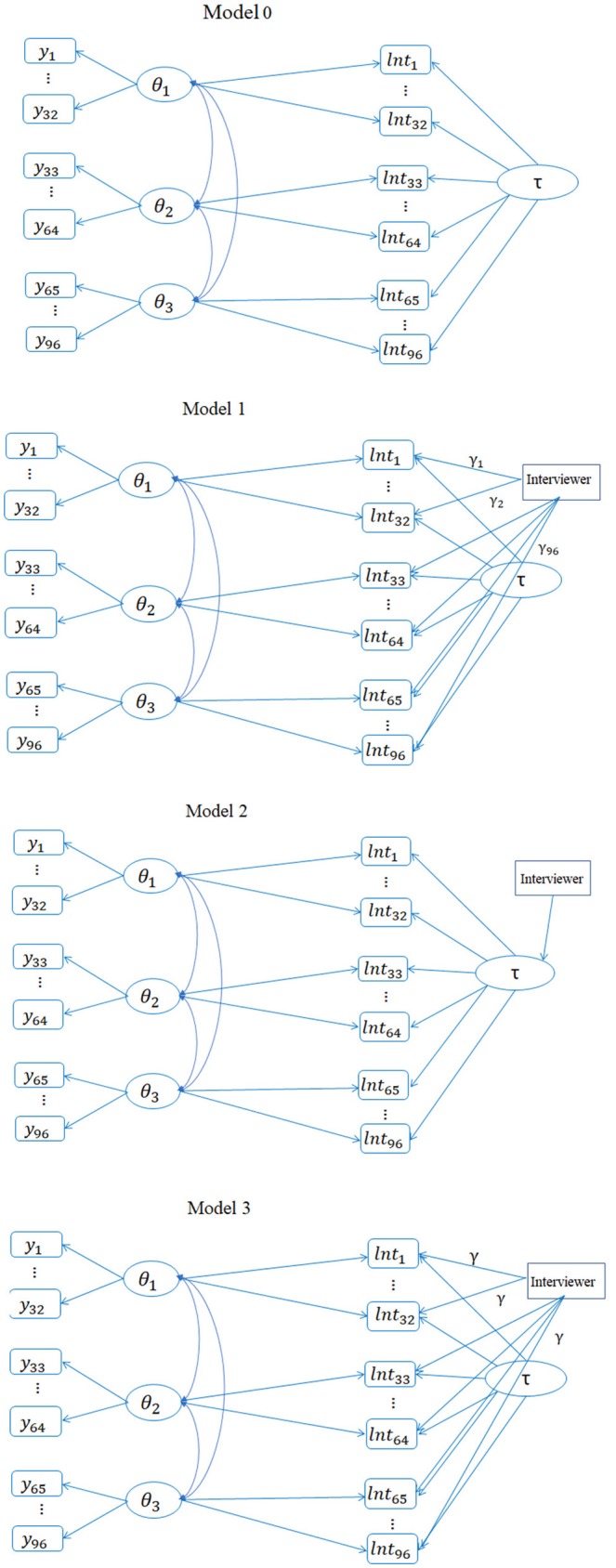
Path diagrams of four different bivariate models (the total number of items is hypothetically 96 for illustration purpose).

The term, φ_*j*_*ρ*_*d*_*θ*_*id*_, is called a cross-relation function (Ranger, 2013; Molenaar et al., 2015), and it is assumed that item *j* measures the *d*th dimension. Different from van der Linden's (2007) hierarchical model in which a covariance structure is assumed on *θ* and τ at a second level, this cross-relation term directly models the relationship between the latent ability and observed log-transformed RTs (log-RTs). Certainly, the cross-relation term based on τ_*i*_ could alternatively enter into the measurement model of responses; for example, Molenaar et al. (2015) argued that incorporating the cross-relation term in the RT model had unique advantages. That is, when the purpose of including RT information is to improve the measurement precision of *θ*, it is preferable to leave the measurement model for the responses unchanged while modeling the information about *θ* (if any) in the RTs. In this regard, *θ* accounts for the shared ability variance in the responses and RTs and τ accounts for the additional, unique variance in the RTs. This joint model is termed as Model 0 and its diagram is shown in Figure 1.

To ensure model identifiability, several constraints need to be in place. First, regarding the MGRM model, the mean and variance of ***θ***s are restricted to be 0 and 1, respectively. Second, the mean and variance of τ is also constrained to be 0 and 1 such that the residual variance of ω_*ij*_ is freely estimated^1^. The three *θ* components are assumed to be correlated, and the correlation matrix is freely estimated. However, all three *θ*s are assumed uncorrelated with τ due to the inclusion of the cross-relation term. The same set of constraints was assumed for all other models introduced hereafter.

Molenaar et al. (2015) suggested identifiability constraints that are similar to those listed, except that var(τ) = 1−*ρ*^2^, instead of 1. Both constraints are sufficient, and their choice conveniently allows the interpretation of *ρ* as a correlation coefficient. Note that in van der Linden's (2007) model the variance of τ is estimable (Equation 22, p. 294). This is because the lognormal model for RT in van der Linden (2007) takes the form

(4) $$f\left(t_{ij};\tau_{i},\alpha_{j},\beta_{j}\right)=\frac{\alpha_{j}}{t_{ij}\sqrt{2\pi }}\exp\left\{-\frac{1}{2}\left[\alpha_{j}\left(\ln\text{​}t_{ij}-(\beta_{j}-\tau_{i})\right)\right]\right\}$$

where α_*j*_ is interpreted as the *dispersion* parameter that quantifies the variance of the lognormal distribution, rather than the *discrimination* parameter as in Equation 3.

### Bivariate Model With Interviewer as a Covariate

Because more than one interviewer was used for data collection, three variations of the bivariate model with interviewer as a covariate were considered. The first model is

(5) $$\ln\text{​}t_{ij}=\lambda_{j}+\;\varphi_{j}\tau_{i}-\;\varphi_{j}\rho_{d}\theta_{id}+\sum_{p=1}^{P}\gamma_{jp}x_{p}+\omega_{ij},$$

where *x*_*p*_ is a binary indicator variable indicating if interviewer *p* recorded the RTs for patient *i*, and *P* is the total number of interviewers in the data. *P* equaled 6 for batch 1 and 5 for batches 2–4. Because each patient interacted with only one interviewer, only one non-zero element in the summation $\overset{P}{\underset{p=1}{\sum }}\gamma_{jp}x_{p}$ enters into the regression equation for patient *i*. The model in Equation 5 (Bivariate Model 1) assumes that interviewer effects differed per item, i.e., there is an interaction between interviewer and items.

Model 2 is a slightly restricted version of Model 1, and the measurement model for RT becomes

(6) $$\ln\text{​}t_{ij}=\lambda_{j}+\;\varphi_{j}\tau_{i}-\;\varphi_{j}\rho_{d}\theta_{id}+\varphi_{j}\sum_{p=1}^{P}\gamma_{p}x_{p}+\omega_{ij},$$

where all parameters have the same interpretations as in Equation 5 except τ_*i*_, which can be interpreted as the individual “residual” speed after removing the interviewer effect. The MGRM model is still used for polytomous responses. In Equation 6, the interviewer effect differs across items but by the same amount, denoted as φ_*j*_. This Model 2 can also be viewed as a hierarchical model in which the interviewer variable predicts the speed at the second level, as follows:

(7) $$\begin{array}{l} \ln\text{​}t_{ij}=\lambda_{j}+\;\varphi_{j}\tau_{i}-\;\varphi_{j}\rho_{d}\theta_{id}+\omega_{ij}\\ \;\tau_{i}=\sum_{p=1}^{P}\gamma_{p}x_{p}+\varepsilon_{i}, \end{array}$$

where ε_*i*_ is the individual residual speed. Compared to Model 1, Model 2 greatly reduces the number of parameters and hence is a more parsimonious model. When fitting the hierarchical model in M*plus* (Muthén and Muthén, 1998-2015), the variance of τ cannot be fixed directly but instead the variance of ε_*i*_ is fixed at 1.

Model 3 considers only the interviewer main effect and it assumes that the interviewer effect does not differ across items. Again the MGRM stays the same, and the model for RTs becomes

(8) $$\begin{array}{r} \ln\;t_{ij}=\lambda_{i}+\;\varphi_{j}\tau_{i}-\;\varphi_{j}\rho_{d}\theta_{id}+\sum_{p=1}^{P}\gamma_{p}x_{ip}+\omega_{ij}. \end{array}$$

Although this Model 3 has essentially the same number of parameters as Model 2, it assumes no interactions between interviewers and items. The path diagrams for the four models are presented in Figure 1.

## Methods

### Instrument and Subjects

Responses and RTs from the Activity Measure for Post-Acute Care (AM-PAC) were analyzed (Yost et al., 2018). The AM-PAC is the first multi-domain patient reported outcomes measure with the capability to direct care in a hospital rehabilitation environment. The scores from the AM-PAC are intended to be linked to the widely understood stages of the Functional Independence Measure (O'Dell et al., 1998; Huang et al., 2000) such that appropriate rehabilitative care plans can be immediately identified. It is anticipated that the AM-PAC will provide an inexpensive and accurate alternative to clinician assessments. The three domains covered in the AM-PAC include Applied Cognition, Daily Activity, and Mobility. A sample question from the Applied Cognition domain is: “*How much difficulty do you currently have reading a long book (over 100 pages) over a number of days*?”, and the four response options are “Unable” (coded as 1), “A lot” (coded as 2), “A little” (coded as 3), and “None” (coded as 4). Items were administered to hospital inpatients via a computer-assisted personal interview using Qualtrics® web survey software. During the field testing of the items, an interviewer read each item to a patient and recorded, on a tablet computer, the patient's responses and the software recorded RTs. A total sample of 2,270 hospitalized patients were recruited to the study; their mean age was 65 years. Roughly 54% were male and 96% were non-Hispanic white, and 78% had two or more comorbidities (Yost et al., 2018).

Questions were grouped into blocks according to domain, and the order of item administration within a block was randomized. Given that there were 324 items in total in the bank, data collection proceeded in four batches to reduce patient burden. The first batch of 109 items was administered to patients, and 24 linking items were selected with eight items in each domain. The number of linking items was determined based on Kolen and Brennan (2004)'s recommendation that at least 20% of the items need to be shared between different test forms to have enough information to link the scale (Wang et al., 2016). These linking items in each domain were selected to produce a composite information function that was closest in shape to the domain information function. Linking items were assembled using the linear programming solver “lp_solve version 5.5” (Diao and van der Linden, 2011). Then, the set of linking items was carried forward in subsequent data collection batches. Table 1 presents the number of items per domain for the four batches.

**Table 1:** Number of unique items per domain for the four batches.

**Batch**	**Applied cognition**	**Daily activity**	**Mobility**	**Total**	
				**Unique**	**Linking**
1	28	27	30	85	24
2	24	24	24	72	24
3	24	24	24	72	24
4	23	23	25	71	24
Linking	8	8	8	24	24
Total	107	106	111	324	—

#### Preliminary Data Cleaning

Table 2 presents the summary descriptive statistics for the four batches of data. The cleaned Batch 1 dataset contained 563 respondents after deleting 67 (10.6%) respondents with at least 20 missing items. The cleaned Batch 2 dataset contained 490 respondents after deleting 52 (9.6%) respondents with more than 10 missing items. The cleaned Batch 3 dataset contained 500 respondents after deleting 55 (9.9%) respondents with more than 9 missing items. The cleaned Batch 4 dataset contained 507 respondents after deleting 36 (6.6%) respondents with more than nine missing items. Although each item contained four response categories, for some items, category 1 and/or category 2 received no responses or very few responses. These items were then recoded to ensure that the lowest response category for each item was always 1, but the highest response category could be 4 or less. As shown in Table 2, the response time distribution exhibited extreme skewness (ranging from 29.08 to 41.84), and therefore the distribution was truncated by removing the top 2.5% and removing the RTs smaller than 3 s, resulting in skewness from 1.48 to 1.66. The resulting data was entered into modeling analysis. Recent research by Marmolejo-Ramos et al. (2015a) suggested that Box-Cox transformation outperformed the elimination method in normalizing positively skewed data. However, the extremely long and short RTs were trimmed in these data because those RTs were considered as outliers. Extremely long RTs happened when the patient took a break such as “service came in to discuss plans” or “patient lunch came and wanted to stop.” The row for the missing proportion of RTs in Table 2 refers to the proportion of RTs at the person-by-item level, out of the cleaned sample size (e.g., 563 for batch 1), that was deleted either because they were extremely short (<3 s) or extremely long (upper 2.5%).

**Table 2:** Descriptive statistics of the observed data, by batch.

**Variable**	**Batch 1**	**Batch 2**	**Batch 3**	**Batch 4**
**SAMPLE SIZE**				
Before cleaning	630	542	555	543
After cleaning	563	490	500	507
Trimmed proportion of RTs	6.24%	3.21%	3.16%	4.59%
**NUMBER OF ITEMS**				
2 categories	1	0	0	0
3 categories	31	26	22	23
4 categories	77	70	94	72
**RT BEFORE TRUNCATION**				
Mean	9.27	9.79	9.82	8.39
SD	21.28	17.39	25.37	17.62
Skewness	41.84	32.40	35.85	29.08
**RT AFTER TRUNCATION**				
Mean	8.21	8.44	8.06	7.18
SD	4.14	4.92	4.80	4.05
Skewness	1.48	1.66	1.65	1.53

To further test the normality of item-level RT distributions, the Kolmogorov-Smirnov (K-S) test (Smirnov, 1948) was conducted for all item-level log-RTs. The K-S statistic quantifies the distance between the empirical distribution function of a sample and the cumulative distribution function of a reference distribution, and it is a non-parametric test of the equality of two distributions. For the present purpose, the K-S test was done with response times that were at least 3 s and were below the 97.5% percentile. This item-level K-S test compared the log-RTs of that item to the theoretical normal distribution with the mean and variance computed for the item. The null hypothesis is that the log-RTs follow a normal distribution. Hence, a significant *p*-value (i.e., *p* < 0.05) indicated that the log-RTs distribution was significantly different from normal. Results showed that in Batch 1, 54 out of 110 items exhibited statistically significant *p*-values, but the K-S statistics for those items were very small (ranged from 0.05 to 0.1). In Batches 2, 3, and 4, 30 out of 96, 16 out of 96, 11 out of 95 items, respectively, had significant *p*-values, but again, the K-S statistics were small.

The K-S test was chosen because of its wide popularity. For instance, it was used to evaluate the item RT distributions from computer-based licensure examinations (Qian et al., 2016). However, other tests, such as the Shapiro-Wilk (S-W) test (Royston, 1982) has been found to be more powerful than the K-S test to detect departure from normality (Marmolejo-Ramos and González-Burgos, 2013). Unsurprisingly, using the S-W tests on the same data set showed that 99.1% of Batch 1 items, 90.6% of Batch 2 items, 95.8% of Batch 3 items and 92.6% of Batch 4 items had significant *p*-values. However, the lognormal model was still used as the parametric model for RTs in the following analysis because the skewness (shown in Table 2) after truncation was not high, and the lognormal distribution was a convenient choice that most software packages can handle.

#### Collapsing Response Categories

In the data analysis, response categories for some items were collapsed due to lack of observations in those categories. Specifically, for a given item, if a category received no response or only one response, the response of this option, if any, was combined into the responses of the next higher category. Therefore, as shown in Table 2, some items had fewer than four response categories. The treatment of collapsing response categories is legitimate for the graded response model because it does not substantially change the item parameter estimates^2^. For instance, a 4-category GRM item (*k* = 1, 2, 3, 4) item will have four parameters, i.e., *a*_*j*_, *b*_*j*1_, *b*_*j*2_, *b*_*j*3_. When collapsing the lowest two response categories, the parameters of the same item become $a_{j}^{*}\approx a_{j}$, $b_{j2}^{*}\approx b_{j2}$, $b_{j3}^{*}\approx b_{j3}$. This is because the GRM is essentially a difference model (see Equation 1), and the same discrimination parameter is assumed across all boundary response functions [i.e., $P_{jk}^{+}\left(\theta \right)$].

### Model Fitting and Item Calibration

#### Bivariate Model Fitting

All four models in Figure 1 were fit with marginal maximum likelihood estimation (MML) using the Expectation-Maximization (EM) algorithm in M*plus*^3^ These models were fitted to each batch of data separately to evaluate global model fit via AIC, BIC and−2Log-likelihood. The M*plus* source code of Batch 4 is provided in the Appendix. The same source code was used for other batches, as well. As shown in Table 3, Model 2 was the best-fitting model across all four batches of data based on BIC, but Model 1 was preferred based on AIC. In addition, Model 2 and Model 3 are respectively nested within Model 1. The deviance test (i.e., likelihood ratio test) revealed that there was a significant difference between Model 1 and Model 2, Model 1 and Model 3, implying that Model 1 should be preferred. However, Model 2 was used in the following analysis for two reasons: (1) Model 2 is a much more parsimonious model than Model 1 and it is conceptually more reasonable because the interviewer effect should not interact with items, i.e., the interviewer's speed should be relatively static across items; (2) when fitting Model 1 in the concurrent calibration described below, it failed to converge due to complexity and data sparsity.

**Table 3:** Global fit results (AIC, BIC,−2Log-likelihood) for the four bivariate models, by batch.

**Batch and model**	**Number of free parameters**	**AIC**	**BIC**	**-2Log-likelihood**
**BATCH 1**				
Model 0	736	133566	136755	132094
Model 1	1281	133174	138725	130612
**Model 2**	**741**	**133316**	**136527**	131834
Model 3	741	133409	136620	131926
**BATCH 2**				
Model 0	652	102468	105202	101164
Model 1	940	102049	105992	100170
**Model 2**	**655**	**102235**	**104982**	100924
Model 3	655	102339	105086	101030
**BATCH 3**				
Model 0	656	111384	114149	110072
Model 1	1040	110613	114996	108532
**Model 2**	**660**	**111001**	**113783**	109682
Model 3	660	111323	114105	110004
**BATCH 4**				
Model 0	648	108550	111290	107254
Model 1	1028	107733	112080	105676
**Model 2**	**652**	**108174**	**110931**	106870
Model 3	652	108364	111121	107060

*Bold values highlighted the best-fitting model based on the information criteria*.

#### Concurrent Calibration

When data are collected in different batches, linking items are used to place the items from the different batches onto a common scale. Concurrent calibration has been demonstrated to be more effective than separate calibration plus *post-hoc* linking (Kolen and Brennan, 2004) because the latter approach suffers from linking error. Three models were compared in the concurrent calibration: the MGRM model for responses only, Model 0, and Model 2. Models 1, and 3 were not considered because of their poorer fit compared to Model 2. Both the item and person parameters and their standard errors were compared across the three models. The main research question was whether including RTs and interviewer information helped improve the estimation accuracy of both item and person parameters.

When pooling data from the four batches together, the concurrent calibration of Model 0 and Model 2 failed to converge due to the sparsity of data and model complexity. Therefore, a two-stage approach was implemented. In the first stage, data from Batches 2–4 were pooled and a concurrent calibration was conducted on the pooled data. Data from Batch 1 was left out because this batch had the largest number of items flagged under the K-S test. By shrinking the sample size, all models successfully converged. Then in the second stage, Batch 1 data were calibrated using the fixed parameter calibration approach (Kim, 2006). That is, the linking item parameters (i.e., *a, b*, λ, and φ) were fixed at their estimated values obtained from Stage 1 for each of the three models such that the remaining items were estimated on the same scale as the linking items. Hence, no further linking procedure was needed.

Due to the collapsing of response categories, a side note for the two-stage approach is worth mentioning. Specifically, for the linking items, the threshold parameters of Batch 1 did not always match those in Batches 2–4. For example, an item had four categories (three threshold parameters) in Batches 2–4, but only three categories (two threshold parameters) in Batch 1. The linking items always had the same or fewer categories in Batch 1 as compared to the combined data due to the smaller sample size of Batch 1. In this case, only the corresponding threshold parameters and discrimination parameter for an item were input into the fixed calibration. The rationale is the same as before—collapsing response categories does not substantially change the item parameters.

## Results

### Global Model Fit

Table 4 presents the global model fit statistics for the three models in both stages. Note that the AIC and BIC from the MGRM are smaller because they are on a different scale compared to Model 0 and Model 2 due to its exclusion of RT information. Consistent with the separate calibration results, Model 2 fit the data better than Model 0, reflected by smaller AIC and BIC values.

**Table 4:** Global model fit results.

**Stage and Model**	**AIC**	**BIC**
**STAGE 1: CONCURRENT CALIBRATION (BATCHES 2-4)**		
MGRM	185303.933	190099.963
Model 0	327447.703	336067.811
**Model 2**	**326589.532**	**335230.884**
**STAGE 2: FIXED PARAMETER CALIBRATION**		
MGRM	74013.471	75391.453
Model 0	134224.604	136824.572
**Model 2**	**133924.461**	**136546.095**

*Bold values highlighted the best-fitting model based on the information criteria*.

### Item Parameter Estimates

**Figure 2:**
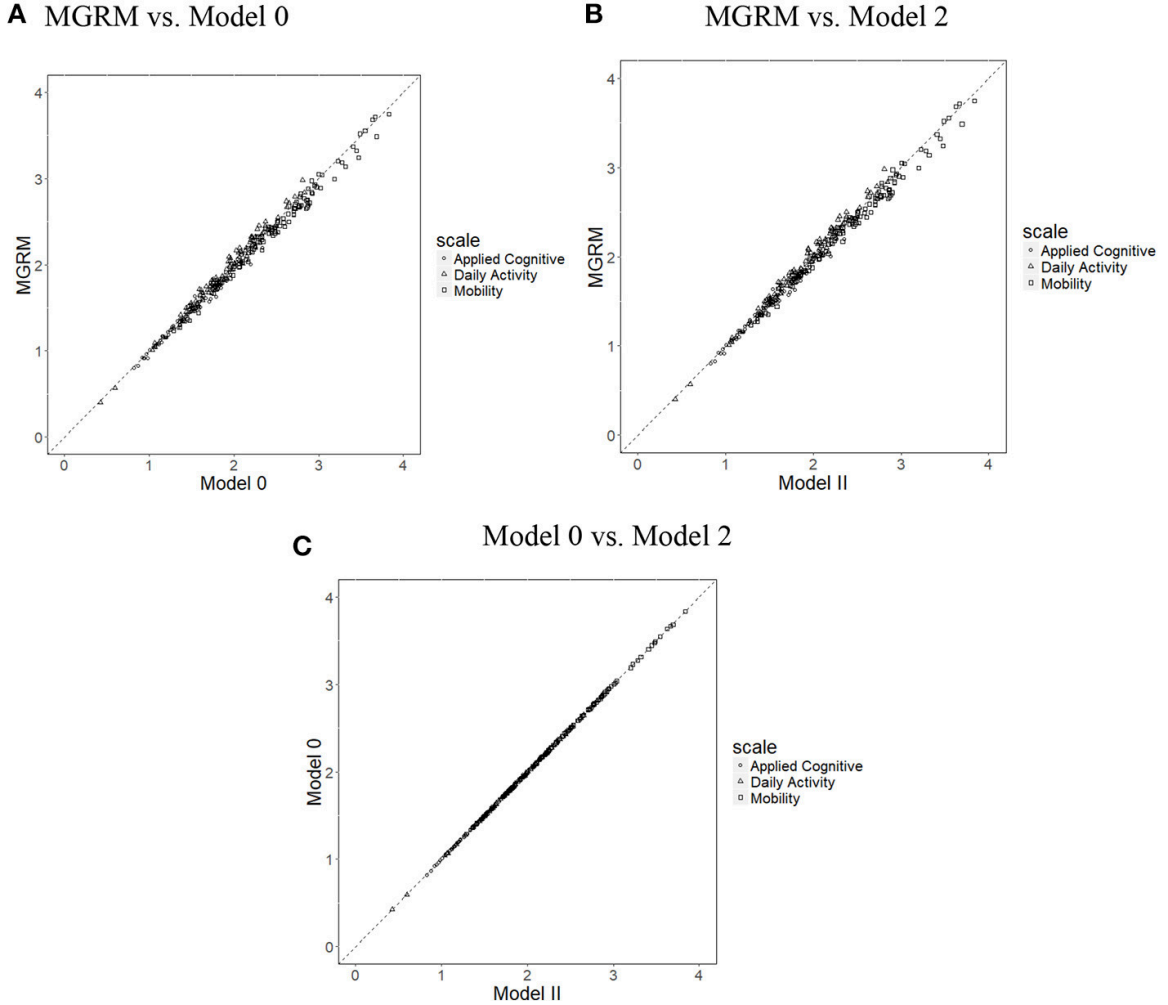
Scatterplots of item discrimination parameters (*a*_*j*_) across three models. **(A)** MGRM vs. Model 0, **(B)** MGRM vs. Model 2, and **(C)** Model 0 vs. Model 2.

Figure 2 presents the scatterplots of item discrimination parameters (*a*_*j*_) across the three models; all points fall along the 45° line, implying a close alignment of item parameter estimates from the three models. This is unsurprising because the variance of *θ* was fixed at 1 across all models, which fixed the scale of *a*_*j*_. Means of SEs of estimates of *a*_*j*_ were 0.188 in the MGRM, 0.190 in Model 0, and 0.190 in Model 2. A simple *t*-test showed no significant differences of SEs between the different models.

**Figure 3:**
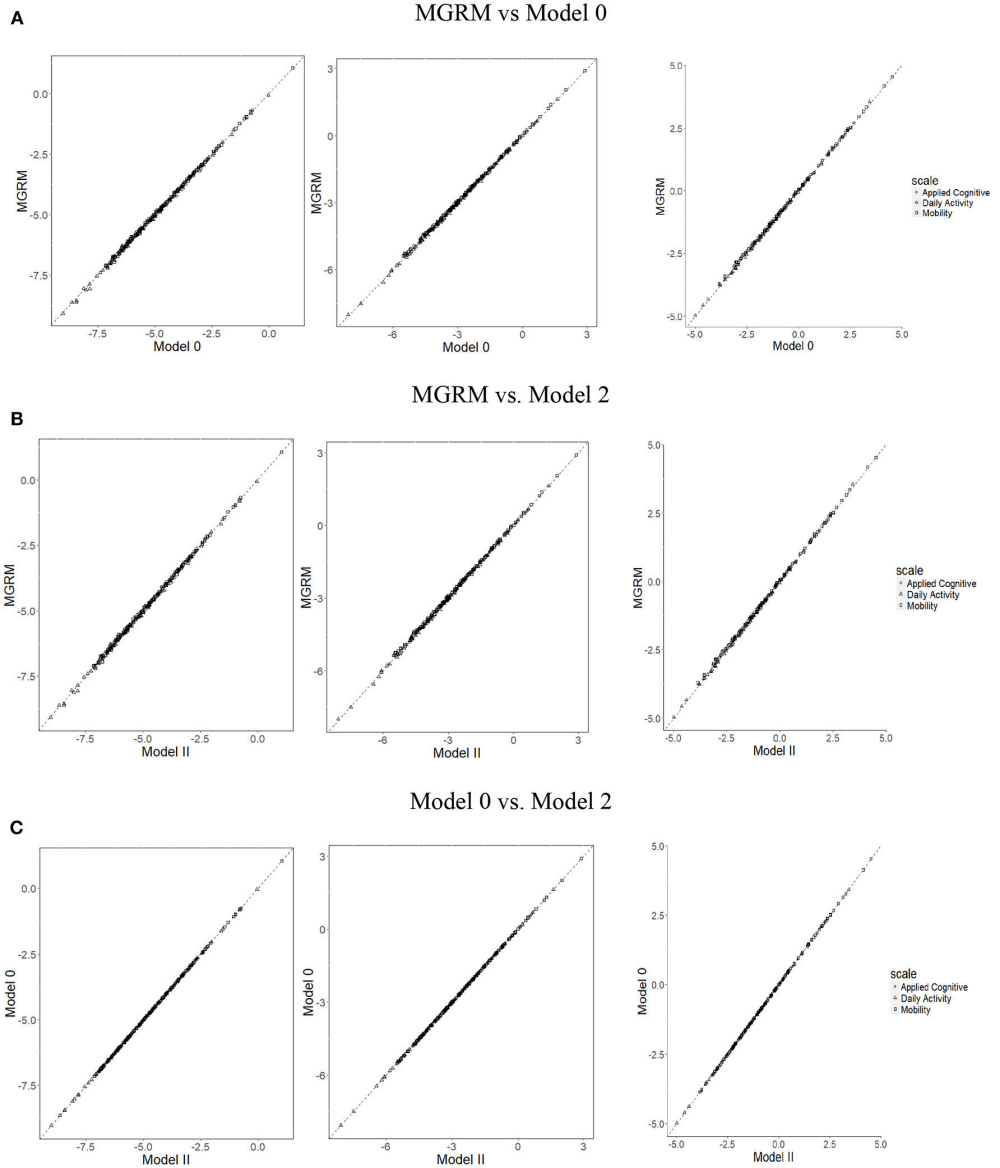
Scatterplots of item boundary parameters (from left to right: ${\hat{b}}_{j1}$, ${\hat{b}}_{j2}$, ${\hat{b}}_{j3}$) across three models. **(A)** MGRM vs. Model 0, **(B)** MGRM vs. Model 2, and **(C)** Model 0 vs. Model 2.

The correlations of boundary parameters ${\hat{b}}_{jk}$ between different models were all 1, and therefore the scatterplots (Figure 3) show that the estimates of ${\hat{b}}_{jk}$ from the different models fall tightly on the 45° line. Moreover, *t*-tests showed no significant differences of mean SEs of ${\hat{b}}_{jk}$ between different models. Thus, the results suggest that, in these data, estimation of MGRM item parameters *a*_*j*_ and *b*_*j*_, and their SEs were not affected by the addition of RT information.

**Figure 4:**
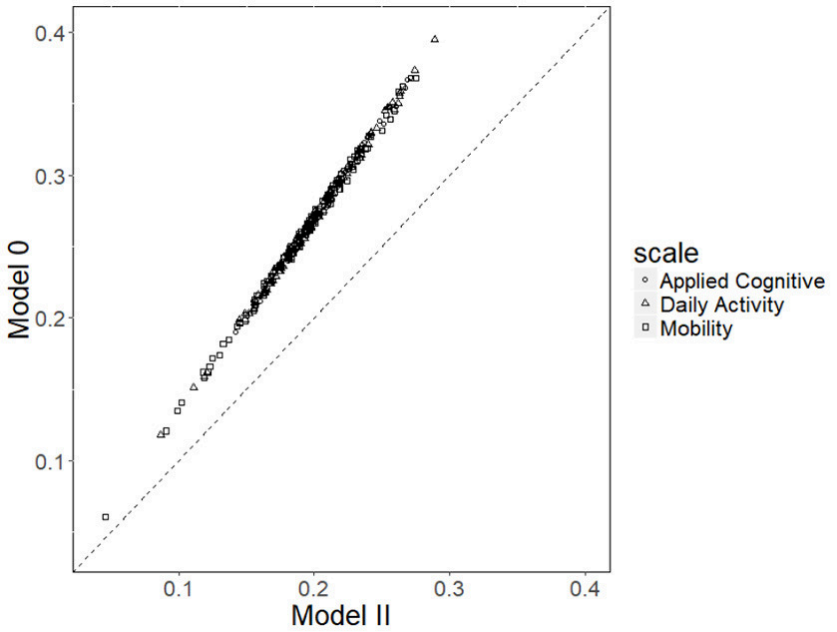
Scatterplot of estimates of ${\hat{\varphi }}_{\text{j}}$ between Model 0 and Model 2.

With respect to the item time discrimination parameter, φ_*j*_, the correlation between their estimates from Model 0 and Model 2 was 0.99. The scatterplot (Figure 4) shows that these estimates of ${\hat{\varphi }}_{j}$ from the two models fell on a line that was not 45°, indicating that there was a linear relationship between ${\hat{\varphi }}_{j}$s from the two models. The explanation is as follows: Focusing on the two terms in Model 0 and Model 2, respectively, φ_*j*_(τ_*i*_− ρ_*d*_*θ*_*id*_) (Equation 3) and $\varphi_{j}\left(\overset{P}{\underset{p=1}{\sum }}\gamma_{p}x_{ip}+\varepsilon_{i}-\;\rho_{d}\theta_{id}\right)$ (Equation 7) the (τ_*i*_− *ρ*_*d*_θ_*id*_) and (ε_*i*_− *ρ*_*d*_*θ*_*id*_) are the same across the two equations because both τ_*i*_ and ε_*i*_ are on the 0–1 scale. Due to the data collection design, the same interviewer went through all items in the batch each time, and each interviewer interviewed a portion of the sample. For instance, suppose the sample size is *N*, and there are *n*_1_, *n*_2_−*n*_1_, *n*_3_−*n*_2_, *n*_4_−*n*_3_, and *N*−*n*_4_ patients interviewed by each interviewer, as shown in Table 5. Then, for item *j*, those patients assigned to Interviewer 1 all carry the same interviewer effect of γ_1_, and similarly for the three other groups. Hence, the second and third columns are the same for every item, and also because the mean and variance of these two columns are different, there is a unique linear relationship between ${\hat{\varphi }}_{j}$s from the two models. On the other hand, SEs of ${\hat{\varphi }}_{j}\;$of Model 2 are significantly lower (*p* < 0.001) than those of Model 0: Mean SE was 0.018 in Model 0 and 0.013 in Model 2.

**Table 5:** An illustration of the linear transformation relationship of ${\hat{\varphi }}_{j}$ from Model 0 and Model 2.

**Patients**	**Model 0 ${\text{φ}}_{\text{j}}^{\text{0}}$**	**Model 2 ${\text{φ}}_{\text{j}}^{\text{2}}$**	**Interviewer**
1 to *n*_1_	(τ_*i*_−*ρ*_*d*_*θ*_*id*_)	(τ_*i*_−*ρ*_*d*_*θ*_*id*_)+γ_1_	1
*n*_1_+1 to *n*_2_	(τ_*i*_−*ρ*_*d*_*θ*_*id*_)	(τ_*i*_−*ρ*_*d*_*θ*_*id*_)+γ_2_	2
*n*_2_+1 to *n*_3_	(τ_*i*_−*ρ*_*d*_*θ*_*id*_)	(τ_*i*_−*ρ*_*d*_*θ*_*id*_)+γ_3_	3
*n*_3_+1 to *n*_4_	(τ_*i*_−*ρ*_*d*_*θ*_*id*_)	(τ_*i*_−*ρ*_*d*_*θ*_*id*_)+γ_4_	4
*n*_4_+1 to N	(τ_*i*_−*ρ*_*d*_*θ*_*id*_)	(τ_*i*_−*ρ*_*d*_*θ*_*id*_)	5 (reference)

Regarding the time intensity parameter, λ_*j*_, again the correlation between their estimates from Model 0 and Model 2 was larger than 0.99. SEs of ${\hat{\lambda }}_{j}$ from Model 2 were significantly higher (*p* < 0.001) than those of Model 0: Mean SE was 0.020 in Model 0 and 0.023 in Model 2. The results suggest that the SE of item response time parameters ${\hat{\varphi }}_{j}$ and ${\hat{\lambda }}_{j}$ is affected in different directions by the addition of interviewers as covariates. However, the absolute difference in SEs was not too large to be concerning because the difference appeared mostly in the third decimal place.

### Person Parameter Estimates

In terms of ***θ*** estimation, the ${\hat{\theta }}_{1}$, ${\hat{\theta }}_{2}$, and ${\hat{\theta }}_{3}$ from all three models correlated as high as 0.99 to 1. Mean SEs from Models 0 and 2 (Table 6) were significantly lower than those from the MGRM (*p* < 0.001), and there was no significant difference of SE between Model 0 and Model 2. This result implies that adding response time decreased the SE of $\hat{\theta }$, which is consistent with prior findings (e.g., van der Linden et al., 2010; Wang et al., 2013a,b), but adding the interviewer variable did not further decrease the SEs.

**Table 6:** Mean and SD of SE of $\hat{\theta }$ from three models.

***θ***	**MGRM**	**Model 0**	**Model 2**
**SE** ***θ***_1_			
Mean	0.307	0.280	0.279
SD	0.093	0.076	0.076
**SE** ***θ***_2_			
Mean	0.252	0.242	0.241
SD	0.082	0.071	0.070
**SE** ***θ***_3_			
Mean	0.178	0.171	0.171
SD	0.079	0.074	0.074

Table 7 presents the estimated correlation parameters. Consistent with previous findings (e.g., Wang et al., 2018a), there were moderate to high correlations among the three latent traits. Moreover, the speed factor also played a modest role as reflected by the moderate size of *ρ*_1_ to *ρ*_3_. These correlations were higher in Model 2 than in Model 0, which is unsurprising because after removing the interviewer effects on RTs, the individual speed factor should correlate higher with individual latent traits.

**Table 7:** Final Pearson correlation parameter estimates for the three models from two calibration stages.

**Stage and Model**	***ρ*_*θ*_1_*θ*_2__**	***ρ*_*θ*_1_*θ*_3__**	***ρ*_*θ*_2_*θ*_3__**	***ρ*_1_**	***ρ*_2_**	***ρ*_3_**	**γ_p_**
**STAGE 1: CONCURRENT CALIBRATION (BATCH 2 TO 4)**							
MGRM	0.624	0.468	0.846	–	–	–	–
Model 0	0.625	0.488	0.839	0.425	0.458	0.418	–
Model 2	0.628	0.492	0.840	0.583	0.629	0.578	(1.150, 1.053, 0.725, −0.911)
**STAGE 2: FIXED PARAMETER CALIBRATION**							
MGRM	0.702	0.545	0.869	–	–	–	–
Model 0	0.707	0.584	0.881	0.400	0.433	0.457	–
Model 2	0.706	0.583	0.880	0.527	0.577	0.605	(1.063, −0.286, 1.315, −0.135, 1.046)

The last column in Table 7 refers to the fixed effects of interviewers. During Batches 2–4 data collection, the same five interviewers were recruited and one of them was randomly selected as the reference for dummy coding. It appears from the estimated ${\hat{\gamma }}_{p}$ that interviewers differed substantially and that is why including the interviewer variable in the model helped improve model data fit. For Batch 1 data collection, a different set of six interviewers was recruited; among them, three overlapped with the other set of five. However, because a different reference interviewer was selected, the estimated ${\hat{\gamma }}_{p}$ from stage 1 and 2 model fitting were not directly comparable. Still, the results show that interviewers operated at different speeds and they contributed to the observed RT variabilities.

## Discussion and Conclusions

Response time as part of the assessment process data has gained great popularity in recent decades in educational and psychological measurement. This is because collecting RTs has become easy, due to computer-based assessment delivery, and RTs provide an additional source of information for researchers to understand an individual's behavior as well as the characteristics of the items. More than a dozen IRT models have been proposed in the psychometrics literature, with an early focus on modeling the different shapes of RT distributions (e.g., Rouder et al., 2003; van der Linden, 2007; Loeys et al., 2011; Wang et al., 2013a,b) and a later focus on modeling within-subject variations such as different and changing test-taking behaviors (e.g., Wang and Xu, 2015; Molenaar et al., 2018; Wang et al., 2018b,c). However, the usage of item-level RT information has rarely been explored in health measurement.

This study systematically investigated the application of RTs for improving measurement precision of the target latent traits and the estimation precision of the item parameters. The bivariate joint model discussed in Molenaar et al. (2015) was applied and expanded in two respects: (1) a multivariate *θ* was considered in the measurement model for responses, and this *θ* vector was correlated with the latent speed through the cross-relation term; (2) an interviewer covariate was entered into the model to explain the variability of the observed RTs. Patient-reported outcomes obtained from personal interview surveys are widely used in health services research studies (Clancy and Collins, 2010), especially when conducting such surveys among older adults or patients with severe symptoms like the sample used in the present research. Thus, the observed RTs might be contaminated by the interviewer's reaction speed and, hence, the interviewer variable should be included in the model.

Several approaches to including the interviewer variable were explored. Results indicated that Model 2, which is a hierarchical model, consistently best fit the data. In this model, the interviewer's effect on the observed RTs is mediated through patients' latent speed. It is more parsimonious than Model 1 in which the interviewer's effect could differ for different items. Indeed, Model 2 also makes more intuitive sense because the interviewer effect reflects the different interviewers' response styles (i.e., fast or slow responders) that could be considered as the latent speed of an interviewer; hence, it should not change from item to item.

Results from the data analysis revealed that (1) adding response time information did not affect the item parameter estimates and their standard errors significantly; and (2) adding response time information helped reduce the standard error of patients' multidimensional latent trait estimates, but adding interviewer as a covariate did not result in further improvement, although the interviewer effect was significant. Regarding the first point, it is not surprising because Ranger (2013) has proven that the amount of (Fisher) information RTs provide to *θ* cannot be >$\frac{\rho^{2}}{1-\rho^{2}}$ (i.e., an upper bound) regardless of test length and RT distributions. A simple explanation is that RTs only contribute to *θ* via τ due to the hierarchical structure in van der Linden (2007), and hence the maximum information RTs provide is when τ is “observed,” resulting in the information upper bound. As a result, the collateral information provided by RT will be useful when test length is short, but its role diminishes in longer tests when information accrued through responses is already high. That said, it is still worth pointing out that the role of speed as a self-contained construct might be useful for psychological and health assessment. It might be particularly promising to investigate the additional validity of the assessment by including speed in the prediction of external criteria.

An immediate implication for the follow-up adaptive design of the AM-PAC is that RT does not need to be included in interim *θ* estimation (i.e., selecting items during assessment delivery), but it could be used to improve the final *θ* estimates. Moreover, to further improve the time efficiency of adaptive testing, the maximum information per time unit (Fan et al., 2012) or its simplified version (Cheng et al., 2017) could be applied. In this case, the interviewer effect could be ignored when estimating an individual patient's speed, as long as item time parameters are provided. This is pragmatically sound because it is likely that different interviewers will be used for adaptive testing data collection in some measurement environments.

Due to the positive skewness of the RT distribution, typical log-transformations were used (van der Linden, 2007; Wang and Xu, 2015; Qian et al., 2016), and the raw RT data was cleaned by trimming the extremely short and long observations. However, recent research by Marmolejo-Ramos et al. (2015a) suggested that the Box-Cox transformation outperformed the elimination methods in normalizing positively skewed data. Vélez et al. (2015) proposed a new approach to estimate the parameter λ in the Box-Cox transformation. In cases in which the log-transformation is insufficient, the Box-Cox transformation could be a viable alternative. In the present study, the extremely long and short RTs were trimmed because those RTs were considered as outliers. On the other hand, when there is lack of information on the outliers, Ueda's method could be used to automatically detect discordant outliers (Marmolejo-Ramos et al., 2015b). Because observed RTs could exhibit different skewed distributions, a careful decision needs to be made with respect to dealing with outliers, data transformation, and using the mean vs. the median, for making valid inferences (Rousselet and Wilcox, 2018). When the median is used, then quantile regression instead of a mean-based linear model should be considered instead.

## Author Contributions

CW contributed to constructing the ideas, explaining the results, and drafting the paper. DW contributed to the constructing the ideas and editing the draft. SS contributed to conducting the data cleaning, model fitting/analysis, and constructing tables/figures.

### Conflict of Interest Statement

The authors declare that the research was conducted in the absence of any commercial or financial relationships that could be construed as a potential conflict of interest.

## Footnotes

^1^By default, M*plus* sets the factor mean to be 0 for both ***θ*** and τ.

^2^A separate study (Jiang and Wang, 2019) was conducted that provided analytic and simulation evidence for this claim.

^3^M*plus* was chosen because it is widely used in social science research. M*plus* plotting using R is available via the “rhdf5” package. For details, refer to http://www.statmodel.com/mplus-R/.
