# Supplementary material for: Modeling Response Time and Responses in Multidimensional Health Measurement
Source: Front Psychol. 2019 Jan 29;10:51. doi: 10.3389/fpsyg.2019.00051 (PMC6361798; doi:10.3389/fpsyg.2019.00051)
Supplement: Supplementary file 1 [file Data_Sheet_1.pdf]

Appendix: M*plus* code with Brief Annotation

```
TITLE: Model I Input File;
  DATA:FILE IS batch_4.txt ;

  VARIABLE:NAMES ARE
   r1-r95 t1-t95 inte1 inte2 inte3 inte4 inte5;
   USEVARIABLES ARE r1-r95 t1-t95 inte1 inte2 inte3 inte4;
   CATEGORICAL ARE r1-r95;
   Missing are all (-99);

  ANALYSIS:
```

Using marginal maximum likelihood estimation (MML) via Expectation-Maximization (EM) algorithm

```
  ESTIMATOR = ML;
  ALGORITHM =EM;

  MODEL:
  abil1 BY r1-r23* r72-r79*(lambda1-lambda23 lambda72-lambda79);
  abil2 BY r24-r46* r80-r87*(lambda24-lambda46 lambda80-lambda87);
  abil3 BY r47-r71* r88-r95*(lambda47-lambda71 lambda88-lambda95);
  speed1 BY t1-t95*(phi1-phi95) ;
  abil1 BY t1-t23 t72-t79*(load1-load23 load72-load79);
  abil2 BY t24-t46 t80-t87*(load24-load46 load80-load87);
  abil3 BY t47-t71 t88-t95*(load47-load71 load88-load95);
  t1-t95 ON inte1* ;   # Adding the interviewer effect
  t1-t95 ON inte2* ;
  t1-t95 ON inte3* ;
  t1-t95 ON inte4* ;
```

For Model II, the above four lines are replaced by
speed1 ON inte1-inte4*;
For Model III, the above four lines are replaced by the following four lines
t1-t95 ON inte1* (1);
t1-t95 ON inte2* (2);
t1-t95 ON inte3* (3);
t1-t95 ON inte4* (4);

The code here onwards are the same for every model

```
  abil1 with speed1@0;
  abil2 with speed1@0;
  abil3 with speed1@0;

  [t1-t95] (icept1-icept95);

  abil1-abil3@1
  speed1@1 ;

  MODEL CONSTRAINT:
```

```
NEW(rho1 rho2 rho3);

load1=-phi1*rho1;
load2=-phi2*rho1;
load3=-phi3*rho1;
load4=-phi4*rho1;
load5=-phi5*rho1;
load6=-phi6*rho1;
load7=-phi7*rho1;
load8=-phi8*rho1;
load9=-phi9*rho1;
load10=-phi10*rho1;
load11=-phi11*rho1;
load12=-phi12*rho1;
load13=-phi13*rho1;
load14=-phi14*rho1;
load15=-phi15*rho1;
load16=-phi16*rho1;
load17=-phi17*rho1;
load18=-phi18*rho1;
load19=-phi19*rho1;
load20=-phi20*rho1;
load21=-phi21*rho1;
load22=-phi22*rho1;
load23=-phi23*rho1;
load24=-phi24*rho2;
load25=-phi25*rho2;
load26=-phi26*rho2;
load27=-phi27*rho2;
load28=-phi28*rho2;
load29=-phi29*rho2;
load30=-phi30*rho2;
load31=-phi31*rho2;
load32=-phi32*rho2;
load33=-phi33*rho2;
load34=-phi34*rho2;
load35=-phi35*rho2;
load36=-phi36*rho2;
load37=-phi37*rho2;
load38=-phi38*rho2;
load39=-phi39*rho2;
load40=-phi40*rho2;
load41=-phi41*rho2;
load42=-phi42*rho2;
load43=-phi43*rho2;
load44=-phi44*rho2;
load45=-phi45*rho2;
load46=-phi46*rho2;
load47=-phi47*rho3;
load48=-phi48*rho3;
load49=-phi49*rho3;
load50=-phi50*rho3;
load51=-phi51*rho3;
load52=-phi52*rho3;
```

```
load53=-phi53*rho3;
load54=-phi54*rho3;
load55=-phi55*rho3;
load56=-phi56*rho3;
load57=-phi57*rho3;
load58=-phi58*rho3;
load59=-phi59*rho3;
load60=-phi60*rho3;
load61=-phi61*rho3;
load62=-phi62*rho3;
load63=-phi63*rho3;
load64=-phi64*rho3;
load65=-phi65*rho3;
load66=-phi66*rho3;
load67=-phi67*rho3;
load68=-phi68*rho3;
load69=-phi69*rho3;
load70=-phi70*rho3;
load71=-phi71*rho3;
load72=-phi72*rho1;
load73=-phi73*rho1;
load74=-phi74*rho1;
load75=-phi75*rho1;
load76=-phi76*rho1;
load77=-phi77*rho1;
load78=-phi78*rho1;
load79=-phi79*rho1;
load80=-phi80*rho2;
load81=-phi81*rho2;
load82=-phi82*rho2;
load83=-phi83*rho2;
load84=-phi84*rho2;
load85=-phi85*rho2;
load86=-phi86*rho2;
load87=-phi87*rho2;
load88=-phi88*rho3;
load89=-phi89*rho3;
load90=-phi90*rho3;
load91=-phi91*rho3;
load92=-phi92*rho3;
load93=-phi93*rho3;
load94=-phi94*rho3;
load95=-phi95*rho3;

OUTPUT:
TECH1, TECH4;
 NOCHISQUARE;
```
